# Antioxidant and Antiacetylcholinesterase Activities of Some Commercial Essential Oils and Their Major Compounds

**DOI:** 10.3390/molecules16097672

**Published:** 2011-09-07

**Authors:** Smail Aazza, Badiâ Lyoussi, Maria G. Miguel

**Affiliations:** 1 Laboratory of Physiology-Pharmacology-Environmental Health, Faculty of Sciences Dhar El Mehraz, BP 1796 Atlas, University Sidi Mohamed Ben Abdallah, Fez 30 000, Morocco; 2 Plant Biotechnology Research Group, Department of Chemistry and Pharmacy, Institute for Biotechnology and Bioengineering, Faculty of Science and Technology, University of Algarve, Campus de Gambelas, 8005-139 Faro, Portugal

**Keywords:** antiacetylcholinesterase, commercial essential oils, DPPH, reductive potential, TBARS

## Abstract

The commercial essential oils of *Citrus aurantium* L., *Cupressus sempervirens* L., *Eucalyptus globulus* Labill., *Foeniculum vulgare* Mill. and *Thymus vulgaris* L., isolated by steam distillation by a company of Morocco were evaluated in terms of *in vitro* antioxidant activity through several methods. *In vitro* acetylcholinesterase inhibitory activity was also determined. *Citrus limon* (L.) Burm. f. oil was also studied, but it was obtained by peel expression. The best antioxidant was *T. vulgaris* oil, independent of the method used, mainly due to the presence of the phenolic monoterpenes thymol and carvacrol, which when studied as single compounds also presented the best activities. Concerning the acetylcholinesterase inhibition activity, *E. globulus* was the most effective. Nevertheless its main components 1,8-cineole and limonene were not the most active, a feature that corresponded to δ-3-carene.

## 1. Introduction

Currently, there is global interest in finding new and safe antioxidants from natural sources, to prevent oxidative deterioration of foods and to minimise oxidative injure of living cells. Antioxidants may act as chemical traps/sinks that “absorb” energy and electrons, quenching ROS (carotenoids, anthocyanidins); catalytic systems that neutralize or divert ROS [antioxidant enzymes SOD (superoxide dismutase), catalase, and glutathione peroxidase]; binding/inactivation of metal ions to prevent generation of ROS (ferritin, ceruloplasmin, catechins); and chain-breaking antioxidants which scavenge and destroy ROS (ascorbic acid, tocopherols, uric acid, glutathione, flavonoids) [1]. Therefore, and based on their mode of action, the antioxidants can be classified as primary or secondary antioxidants. Primary antioxidants are able to donate a hydrogen atom rapidly to a lipid radical, forming a new, more stable radical. Secondary antioxidants react with the initiating radicals (or inhibit the initiating enzymes), or reduce the oxygen level (without generating reactive radical species). Therefore, these secondary antioxidants can retard the rate of radical initiation reaction by elimination of initiators [2]. Since antioxidants can act through several mechanisms, the detection of such activity must be evaluated using various assays. *In vitro*, antioxidant assays in foods and biological systems can be divided in two groups: those that evaluate lipid peroxidation and those that measure free radical scavenging ability [3].

Essential oils contain volatile aroma compounds from aromatic plants. They are complex mixtures of compounds belonging to diverse chemical families (terpenes, alcohols, aldehydes, phenolic compounds, esters, ethers, ketones). There are several reports regarding the antioxidant activity of the essential oils measured through diverse methods. Miguel [2,3] briefly reviewed the antioxidant ability of several essential oils extracted from aromatic plants, as well as some factors that can influence the chemical composition of the oils and consequently the biological activity, although the correlation between the biological activity (*i.e.*, the antioxidant activity) of essential oils and their chemical composition is often very complicated [2,3]. There are two main approaches to determining the *in vitro *antioxidant activity of essential oils:

(1). The inhibition of lipid oxidation in different systems (oils, solutions of lipids in organic solvents, oil-in-water emulsions, micelles, liposomes, *etc.*);
(2). The ability to scavenge free radical species [3].

Acetylcholinesterase (AChE) is the principal enzyme involved in the hydrolysis of acetylcholine (ACh). According to the references cited in Fujiwara *et al.* [4], the great reduction of this neurotransmissor in the cerebral cortex is a significant factor in Alzheimer’s disease. Since ancient times phytochemicals have been used in the Chinese and Ayurvedic cultures to restore and declining cognitive functions lost with progression of Alzheimer’s disease [5]. Many essential oils and their monoterpenes have been also investigated for their capacity of inhibiting AChE. For example, studies concerning the AChE inhibitory activity and chemical composition of commercial essential oils performed by Dohi *et al.* [6] demonstrated for the first time that eugenol was a potent AChE inhibitor.

Recently, a study concerning the AChE inhibitory of *Thymus* essential oils from Portugal was reported [7]. Such results proved the diversity of results depending on the chemical composition.

In the present work, the essential oils of *Citrus aurantium* L., *Cupressus sempervirens* L., *Eucalyptus globulus* Labil., *Foeniculum vulgare* Mill. and *Thymus vulgaris* L., isolated by steam distillation by a company of Morocco were evaluated in terms of *in vitro* antioxidant activity through several methods. Their *in vitro* acetylcholinesterase inhibitory activity was also determined. *Citrus limon* (L.) Burm. f. oil was also studied, but it was obtained by peel expression. Some of the main components of the oils were used as standards for comparison with the whole oil. Such a study is the continuation of our research on essential oils with antioxidant activity. In this case, such a search is allied with the interests of the Moroccan company to know much more about the potentialities of their essential oils obtained from those species.

Preparations from peel, flowers and leaves of *Citrus aurantium* L. are popularly used for minimizing central nervous system disorders, anxiety treatment, sedative and for treating gastritis and gastric disorders [8,9]. Essential oils and the monoterpene limonene are also largely used as flavouring agents in some foods [10].

In the traditional systems of Indian medicine, the fruit peel of *Citrus limon* (L.) Burm.f. (Rutaceae) is used as a stomachic, carminative, diaphoretic, astringent, febrifuge, and diuretic agent. It regulates skin moisture, softens hard and rough skin, and has a cleaning effect on the oily skin [11,12]. The most common usage of essential oil of lemon is in the treatment of throat infections and in beverages. Together with other plant essential oils, lemon oil was described to treat snoring when used either as an essential oil spray or as a gargle formulation [13].

*Cupressus sempervirens *L. (Cupressaceae) possesses biological properties, acting as expectorant, antipyretic, diaphoreric, and urine enhancer; externally it has been used for treating coughs and bronchitis, for haemorrhoids and against foot sweating. This plant has been also used as an antibacterial and antifungal [14,15].

*Eucalyptus globulus* (Myrtaceae) has been used in folk medicine all over the world for its antibacterial, antifungal, analgesic and anti-inflammatory properties [16,17].

Several parts of *Foeniculum vulgare* Mill. (fennel; Umbelliferae/Apiaceae) are used as a secretomotor, secretolytic, antiseptic, expectorant, spasmolytic, carminative and galactagogue [18].

*Thymus vulgaris* L. (thyme) (Lamiaceae) acts as an expectorant and spasmolytic agent for the bronchia. It has been also used in culinary as an aromatic constituent for seasoning various dishes and as a preservative for foods [19].

## 2. Results and Discussion

### 2.1. Main Components Present in the Essential Oils

The main components detected in the essential oils are presented in Table 1. A detailed discussion of the composition is not presented here because the main purpose of this work was to evaluate the antioxidant and inhibition of acetylcholinesterase activities of the samples as well as those of the main components detected in these commercial essential oils.

**Table 1:** Main components of the essential oil (%).

Components	RI	*T. vulgaris*	*C. aurantium*	*C. sempervirens*	*E. globulus*	*C. limon*	*F. vulgare*
α-Pinene	930			49			
δ-3-Carene	1000			18			
*p*-Cymene	1003	24					
1,8-Cineole	1005				38		
Limonene	1009			32	55	99	
Linalool	1074		59				
Borneol	1134	16					
Linalyl acetate	1245		23				
*trans*-Anethole	1254						75
Thymol	1275	12					
Carvacrol	1286	16					

RI: Retention Index relative to C_9_–C_21_* n-*alkanes on the DB1 column. Empty spaces may means absence of the compounds or with concentrations <10%.

### 2.2. Antioxidant Activities of Essential Oils and Standards

#### 2.2.1. Lipid Peroxidation Inhibition by the Thiobarbituric Acid Reactive Species (TBARS) Method

**Figure 1:**
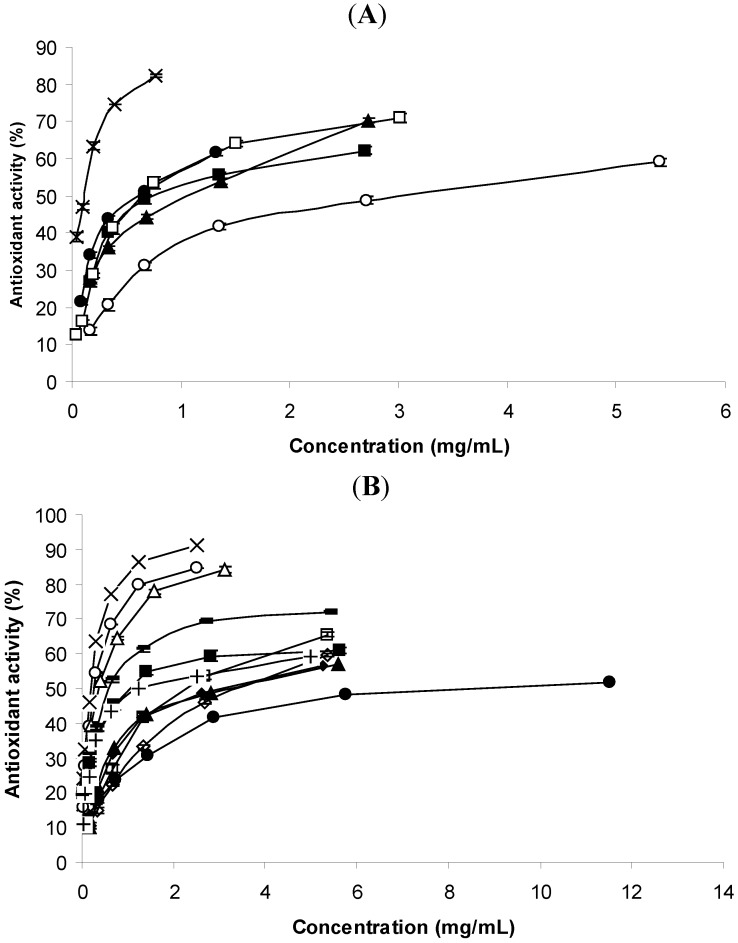
(**A**). TBARS percentage antioxidant indices of the essential oils. (x) *T. vulgaris*; (·) *C. aurantium*; (■) *C. sempervirens*; (▲) *E. globulus*; (○) *C. limon*; (□) *F. vulgare*.; (**B**). TBARS percentage antioxidant indices of the standards. (x) Thymol; (○) Carvacrol; (Δ) *trans*-Anethole; (◊) α-Pinene; (□) *p*-Cymene; (■) Linalool; (▲) Linalyl acetate; (·) 1,8-Cineole; (♦) Limonene; (+) Borneol; (−) δ-3-Carene.

This method measures the malondialdehyde (MDA) formed after lipid hydroperoxide decomposition, which forms a pink chromophore with thiobarbituric acid (TBA). This coloured complex, which absorbs at 532 nm, results from the condensation of two equivalents of TBA and one equivalent of malondialdehyde in an acidic environment [20,21]. The antioxidant activity of all essential oils was dose dependent (Figure 1A). In addition, all of them attained a plateau, beyond which higher concentrations of samples did not improve the antioxidant activity (Figure 1A).

*T. vulgaris* oil presented the best antioxidant activity, since lower concentrations showed higher percentages of oxidation inhibition (Figure 1A). More than 80% inhibition was observed with 0.8 mg/mL of *T. vulgaris* oil, when it reached the plateau. None of the remaining oils was able to reach such an inhibition capacity.

In contrast to *T. vulgaris* oil, *C. limon* oil was the worst for preventing the lipid peroxidation, as 5.4 mg/mL of such sample only presented 59% of activity. Between these *T. vulgaris* and *C. limon* oils are the *C. sempervirens*, *C. aurantium* and *F. vulgare* oils, with slight differences among them. Between *E. globulus* and *C. sempervirens* oils, *E. globulus* oil at lower concentrations was the worst for preventing the formation on malonaldehyde, nevertheless for higher concentrations (2.6 mg/mL) it presented higher inhibition percentage than that of *C. sempervirens* (Figure 1A).

*F. vulgare* oil at lower concentration had similar percentages of antioxidant activities to those of *C. aurantium*, but was superior at higher concentrations (Figure 1A). The statistical treatment of the IC_50_ values (concentrations of the volatile oils able to prevent 50% of lipid oxidation) of these oils proved that *T. vulgaris* (IC_50_ = 0.116 mg/mL) was significantly better than the remaining essential oils, in contrast to the *C. limon* oil (Table 2). There were no significant differences between *C. aurantium* (IC_50_ = 0.652 mg/mL) and *F. vulgare* oil (IC_50_ = 0.652 mg/mL), being better as antioxidants than *E. globulus* (IC_50_ = 1.109).

Although lipid peroxidation was evaluated by a different method (the β-carotene bleaching test) some authors [22] also observed that *T. vulgaris* oil possessed the best activity, even better than the synthetic antioxidant BHA. The same authors obtained similar percentages of inhibition for *C. sempervirens* and *E. globulus* oils, somewhat different of our results, in which *C. sempervirens* had better antioxidant ability than *E. globulus* (Table 2). According to the same authors, the composition of *T. vulgaris* oil also showed *p*-cymene (15.3%), carvacrol (7.96%) and thymol (6.84%) in relative high percentages.

The best activity of *T. vulgaris* may be explained by the presence of thymol and carvacrol, two phenolic compounds with known antioxidant activity [23]. When we tested the antioxidant activity of some of the major compounds of the essential oils, the good activity of both compounds was confirmed (Figure 1B, Table 2). Along with these two standards, *trans*-anethole was also effective as antioxidant with a relative low IC_50_ value (Table 2). These three compounds were the only ones which had IC_50_ < 0.5 mg/mL. *F. vulgare* oil, predominantly constituted by *trans*-anethole (75%), also had a good antioxidant activity (IC_50_ = 0.652 mg/mL), but analogous to that of *C. aurantium*, in which linalool predominated.

**Table 2:** Antioxidant and antiacethylcholinesterase activities (IC_50_ = mg/mL) of the essential oils and standards.

Plant or standard	TBARS	DPPH	AChE inhibitory activity
	IC_50_ (mg/mL)	IC_50_ (mg/mL)	IC_50_ (mg/mL)
*T. vulgaris*	0.116 ± 0.125 ^i^	0.259 ± 0.476 ^d^	0.2169 ± 0.0115 ^f^
*C. aurantium*	0.652 ± 0.125 ^gh^	4.786 ± 0.476 ^c^	nd
*C. sempervirens*	0.766 ± 0.125 ^fg^	8.245 ± 0.476 ^b^	0.2837 ± 0.0115 ^e^
*C. limon*	3.193 ± 0.125 ^c^	16.145 ± 0.476 ^a^	0.8499 ± 0.0115 ^c^
*E. globulus*	1.109 ± 0.125 ^f^		0.1298 ± 0.0115 ^g^
*F. vulgare*	0.652 ± 0.125 ^gh^		1.1877 ± 0.0115 ^b^
Thymol	0.172 ± 0.125 ^i^	0.051 ± 0.476 ^d^	0.2124 ± 0.0115 ^f^
Carvacrol	0.267 ± 0.125 ^hi^	0.052 ± 0.476 ^d^	0.0917 ± 0.0115 ^h^
*trans*-Anethole	0.354 ± 0.125 ^ghi^	-	1.3244 ± 0.0115 ^a^
α-Pinene	3.715 ± 0.125 ^b^	-	nd
*p*-Cymene	2.313 ± 0.125 ^d^	-	nd
Linalool	1.059 ± 0.125 ^f^	-	nd
Linalyl acetate	3.421 ± 0.125 ^bc^	-	nd
1,8-Cineole	9.360 ± 0.125 ^a^	-	0.1082 ± 0.0115 ^gh^
Limonene	3.346 ± 0.125 ^bc^	-	0.5863 ± 0.0115 ^d^
Borneol	1.689 ± 0.125 ^e^	-	0.1321 ± 0.0115 ^g^
δ-3-Carene	0.603 ± 0.125 ^gh^	-	0.0358 ± 0.0115 ^i^

-: It was impossible to determine IC_50_ values, because the highest volume usable in the experiment did not reach 50% of DPPH scavenging (Table 3); nd: not determined.

Two cultivars of fennel with high levels of *trans*-anethole showed remarkably elevated antioxidant activity compared to another cultivar in which estragole predominated [24]. These authors suggested that the double bond of the propenyl side chain in *trans*-anethole conjugated with the aromatic ring can easily form a conjugated radical cation that can be easily delocalized with this aromatic ring and further stabilized by the methoxy group through the 1,4 interaction. The structure of estragole does not present that double bond conjugated with the aromatic ring and therefore can only form a homo-benzyllic radical cation.

δ-3-Carene was the fourth best standard as antioxidant (IC_50_ = 0.603 mg/mL), which can partly explain the relative good activity of *C. sempervirens* oil (0.766 mg/mL). The relatively good activity of that standard is not supported by the results reported by Ruberto and Baratta [23]. Linalool was the fifth best antioxidant (IC_50_ = 1.059 mg/mL), which can partly explain the relative good activity of *C. aurantium* oil (IC_50_ = 0.652 mg/mL). The result of the standard is not supported by those reported by Ruberto and Baratta [23], who using the same method only found a pro-oxidant activity for linalool.

1,8-Cineole, the standard with the highest IC_50_ = 9.360 mg/mL, therefore the worst antioxidant, was also considered a poor antioxidant in [23], since 1 g/L only had 20% of inhibition, a value close to our results (Figure 1B). *T. mastichina* oil in which 1,8-cineole dominated, was revealed to be ineffective as an antioxidant when the activity was measured by the same method [25]. In the presence of the radical inducer 2,20-azobis-(2-amidinopropane) dihydrochloride (ABAP) it had pro-oxidant activity at some of the concentrations checked. Nevertheless, *E. globulus* oil (IC_50_ = 1.109 mg/mL) in which 1,8-cineole (38%) was one of the most important components, along with limonene (55%), did not show as low antioxidant activity as the 1,8-cineole standard. The presence of limonene could explain the better activity of the essential oil, nevertheless its IC_50_ = 3.346 mg/mL is also superior to that observed for the essential oil of *E. globulus*. Only synergistic effects between these two compounds could explain the antioxidant activity of *E. globulus* oil, since 93% of the oil is constituted by 1,8-cineole and limonene. *C. limon*, practically constituted solely by limonene (99%) was the least effective oil as antioxidant. The sample and standard possessed practically similar IC_50_ values, which can explain the worst activity of *C. limon *(Table 2).

#### 2.2.2. Ability for Scavenging DPPH Free Radicals

**Figure 2:**
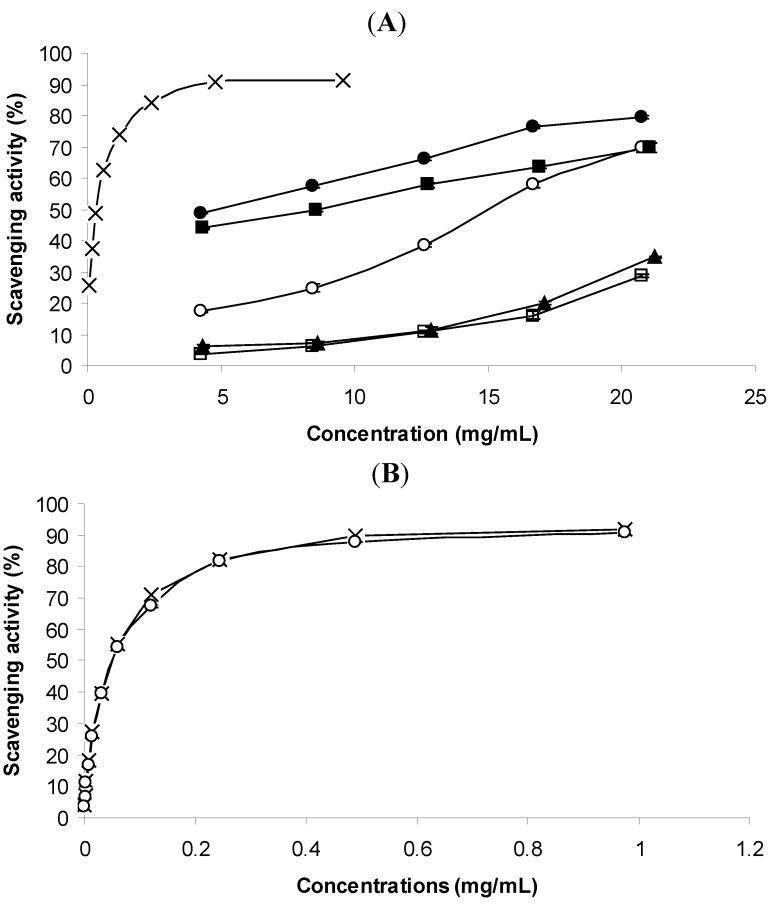
(**A**). DPPH free radical scavenging activity of the essential oils. (x) *T. vulgaris*; (·) *C. aurantium*; (■) *C. sempervirens*; (▲) *E. globulus*; (○) *C. limon*; (□) *F. vulgare*; (**B**). DPPH free radical scavenging activity of the standards. (x) Thymol; (○) Carvacrol.

The reduction ability of DPPH radicals formation was determined by the decrease in its absorbance at 517 nm induced by antioxidants. DPPH is a stable free radical and accepts an electron or hydrogen radical to become a stable diamagnetic molecule [26]. Figure 2A depicts the DPPH scavenging activity of the essential oils. The capacity for scavenging DPPH free radicals was also dose-dependent as already reported for the ability for preventing lipid oxidation. *T. vulgaris* also presented the best activity as already seen for the prevention of malondialdehyde formation (Figure 1A). The difference between this oil and the remaining ones was remarkable. This activity can be attributed to the presence of the phenolic compounds thymol and carvacrol (Figure 2B). The scavenging activity of these two phenolic monoterpenes present in *Thymus* essential oils or as standards was already reported by several authors [23,24,25,26,27,28], although some of them had found differences between the activities of carvacrol and thymol [23,29]. In our case and for DPPH scavenging activity such differences were not observed (Figure 2B).

The essential oils of *E. globulus* and *F. vulgare* were the worst at scavenging DPPH free radicals. Even at higher concentrations, they were not able to scavenge 50% of free radicals (IC_50_ values). The same was found for their main components that at the highest concentration that can be used in the method (the highest volume) were not able to scavenge 50% of DPPH free radicals (Figure 2, Table 2 and Table 3).

**Table 3:** Antioxidant activities measured by two methods and AChE inhibitory activity, in which it was not possible to determine IC_50_ values (DPPH and AChE inhibitory activity) or to make curves (reductive potential) due to the relative low activities of samples.

Plant or standard	DPPH	Reductive potential
	Scavenging activity (%)	Absorbance (770 nm) ^a^
*E. globulus*	34.8 ± 0.2	
*F. vulgare*	28.8 ± 0.4	
*trans*-Anethole	39.8 ± 0.3	
α-Pinene	40 ± 0.2	0.034 ± 0.002
*p*-Cymene	36.7 ± 0.3	0.090 ± 0.006
Linalool	38.2 ± 0.2	0.052 ± 0.005
Linalyl acetate	39.1 ± 0.2	0.050 ± 0.002
1,8-Cineole	36.3 ± 0.3	0.068 ± 0.002
Limonene	38.2 ± 0.5	
α-Borneol	19.6 ± 1.1	0.025 ± 0.002
δ-3-Carene	21.1 ± 0.5	

**Figure 3:**
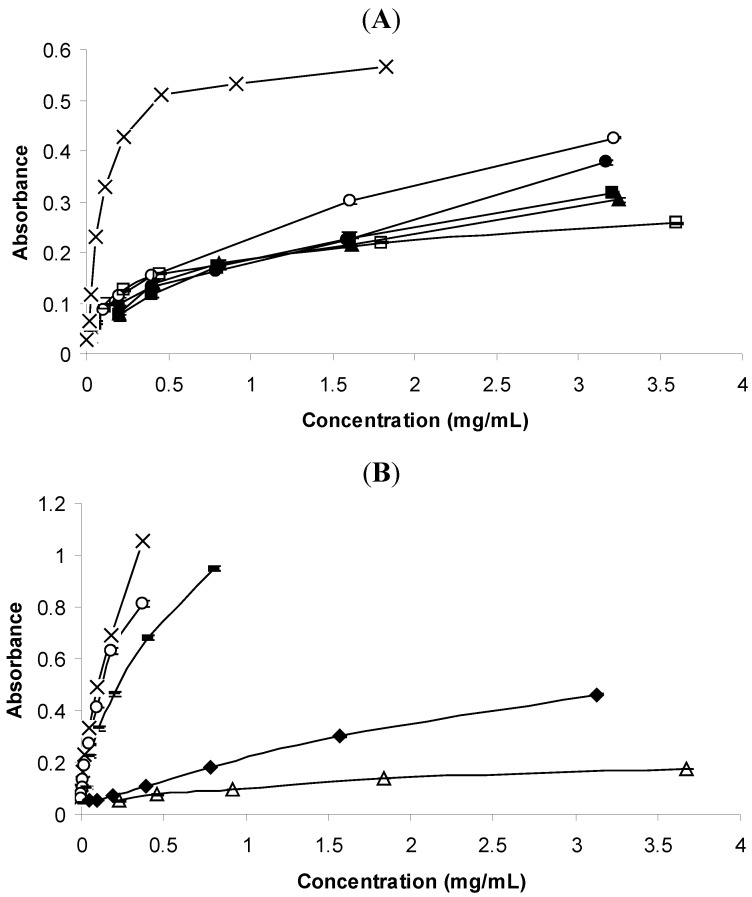
(**A**). Reductive potential of the essential oils. (x) *T. vulgaris*; (·) *C. aurantium*; (■) *C. sempervirens*; (▲) *E. globulus*; (○) *C. limon*; (□) *F. vulgare*; (**B**). Reductive potential of the standards. (x) Thymol; (○) Carvacrol; (Δ) *trans*-Anethole; (♦) Limonene; (−) δ-3-Carene.

±: standard deviation; ^a^ For the blank spaces there are curves which must be seen in Figures 3A and 3B.

The main compounds found in *E. globulus* and *F. vulgare* oils were 1,8-cineole (38%) and limonene (55%); and *trans*-anethole (75%), respectively. The highest percentages of DPPH scavenging found ranged from 36% for 1,8-cineole, to 40% for *trans*-anethole. Therefore the poor activity of the oils may be attributed to the weak ability of their main components to scavenge DPPH free radicals. Although some authors had been able to evaluate the IC_50_ for the *trans*-anethole-rich fennel cultivars, they were significantly different (lower) to those found when TBARS was used as a method for evaluating the antioxidant activity. In our experiment, we also found lower activity which may be explained by the possible different mechanisms involved in the assays. All samples, except the standards thymol and carvacrol, had lower IC_50_values when measured by the DPPH method than with the TBARS method (Table 2). Some authors have criticised the scarce specificity of the thiobarbituric acid method because it seems to give over-estimated results [30,31,32]. This seems having occurred in our samples, because better results were obtained with the TBARS method than the DPPH method, nevertheless, concerning the standards thymol and carvacrol, this was not valid. Therefore, other factors must be responsible for this discrepancy of results.

Among the essential oils studied, lemon oil possessed the weakest capacity for scavenging radicals. This oil is practically constituted solely by limonene (99%). It is noteworthy to stress the difference between the limonene standard and the lemon oil. Limonene had weak ability for scavenging DPPH radicals, which did not allow determination of its IC_50_, and the lemon oil, mainly constituted by limonene, although poor at scavenging free radicals when compared to the remaining essential oils, it was able nevertheless able to scavenge at least 50% of DPPH radicals.

Linalool (59%) and linalyl acetate (23%) constitute the major part of the essential oil of *C. aurantium*. In terms of antioxidant activity it was the second best, and none of the major compounds were phenols. However it is important to mention that this activity was significantly inferior to that observed for *T. vulgaris* (Figure 2A and Table 2). By measuring the formation of hydroperoxydienes from linoleic acid in a micellar system in which the capacity to give a hydrogen atom to stop the radical chain is probably one of the most important mechanisms of this reaction, Ruberto and Baratta [23] reported that linalool had no activity. Through the method of TBARS, such a compound had a pro-oxidant effect. In our case, and considering the mechanism of DPPH method is closer to the micellar system described by Ruberto and Baratta [23], the presence of linalool in the *C. aurantium* oil did not negatively interfere with the capacity for scavenging free radicals.

In contrast to the samples, the standards of thymol and carvacrol were significantly better at scavenging DPPH free radicals than preventing lipid oxidation. Ruberto and Baratta [23] reported that these phenolic compounds were better for avoiding the formation of hydroperoxydienes, that is, the first step of the degradation process of a lipid matrix, whose final products are hydroperoxydienes; than preventing the formation of malondialdehyde, one of the secondary lipid peroxidation products, whose quantification provides a measure of the extent of lipid degradation.

#### 2.2.3. Reductive Potential

Fe(III) reduction can be used as an indicator of electron-donating activity and therefore reflects an important mechanism of phenolic antioxidant action. In this study, the reducing power was evaluated by monitoring the ferric-ferrous transformation at 700 nm. The reducing ability generally increased with increasing sample concentration [33]. *T. vulgaris* oil had the best significant reductive potential, in contrast to that of *F. vulgare* oil. Nevertheless it is noteworthy to stress the great difference between the oil of *T. vulgaris* and the remaining essential oils, as already seen for the other methods used. There was also a dose-dependent behaviour, although it was much more evident in *T. vulgaris* oil.

An unexpected behaviour was found for *C. limon* oil, which had the second best reductive potential in this test. This may be attributed to the limonene since the standard also showed better activity than *trans*-anethole, the main component of the *F. vulgare* oil, which had the worst reductive potential.

Thymol and carvacrol continued to be the standards with the best activities in this assay, along with δ-3-carene. This ability of this standard was unexpected. The assay conditions may have been responsible for the autoxidation of δ-3-carene [34,35], which is not related to the antioxidant ability.

The highest concentration usable in the assay of the remaining standards had a very low absorbance that did not permit us to plot in the corresponding graphic. Lower amounts did not absorb at the wavelength of 770 nm. Therefore the values of absorbance at the highest concentrations used are depicted in Table 3.

For *T. capitata* oil, the results showed an evident positive correlation between DPPH radical scavenging ability and the reducing power assay, which indicated that the reducing ability of the *T. vulgaris* oils contributed in part to the antioxidant activity. Therefore such reducing ability may be attributed to the phenolic compounds thymol and carvacrol, which had also good DPPH scavenging ability and reducing power (Figure 2B and Figure 3B).

The relative good antioxidant activity of *F. vulgare* oil and its main component *trans*-anethole in the TBARS method and the weak antioxidant activity of the same oil detected in the DPPH method and in the reductive potential, suggest that this essential oil act prevented the degradation of hydroperoxides of a lipid system into their secondary oxidation products, therefore avoiding the formation of malondialdehyde. However, the mechanism seems not to involve a reducing ability for preventing the formation of the secondary oxidation products. The positive correlation between the DPPH method and reductive potential was already reported in [33] for *Thymbra capitata* oil, mainly constituted by carvacrol. Such result may confirm the mechanism involved in the antioxidant activity of the thymol and/or carvacrol-rich oils.

### 2.3. Acetylcholinesterase (AChE) Inhibitory Activity

Acetylcholine (ACh) is one of the major compounds by which nerve impulses are transmitted from nerve cell to nerve cell or involuntary muscles. At the cholinergic synapses, acetylcholinesterase (AChE) rapidly breakdowns ACh into choline and acetate. AChE therefore regulates nerve impulse transmission across cholinergic synapses [36]. Inhibition of AChE has been considered as a promising strategy for the treatment of neurological disorders such as Alzheimer’s disease, senile dementia, ataxia and myasthenia gravis, in which a deficit in cholinergic neurotransmission is involved [37]. Potential AChE inhibitors isolated from plant sources have been studied, including essential oils [6,37].

**Figure 4:**
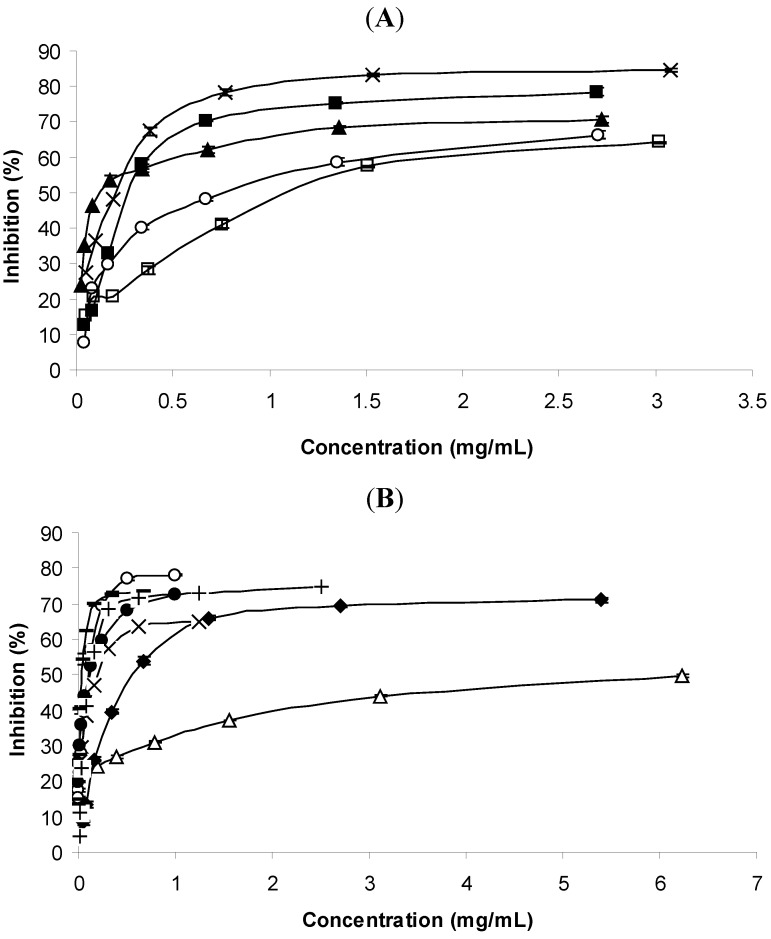
(**A**). Acetylcholinesterase inhibition activity of the essential oils. (x) *T. vulgaris*; (■) *C. sempervirens*; (▲) *E. globulus*; (○) *C. limon*; (□) *F. vulgare*; (**B**). Acetylcholinesterase inhibition activity of standards. (x) Thymol; (○) Carvacrol; (Δ) *trans*-Anethole; (·) 1,8-Cineole; (♦) Limonene; (+) Borneol; (−) δ-3-Carene.

Figures 4A and 4B depict the AChE inhibitory activities of the essential oils and some of their main components at different concentrations. In both cases, the activities were dose-dependent. Lower concentrations of *E. globulus* oil were needed for inhibiting at least 50% the AChE activity, whereas the remaining oils needed more elevated concentrations. This was also confirmed when IC_50_ values were determined: 0.1298 mg/mL for *E. globulus*, significantly inferior to the remaining oils (Table 2). Nevertheless higher percentages of enzyme inhibition were obtained with higher concentrations of *T. vulgaris* and *C. sempervirens* oils, in contrast to the *E. globulus* which reached the plateau with lower concentrations (Figure 4A). The poorest activities were obtained with the oils of *C. limon* and *F. vulgare*. The quantity of the *C. aurantium* oil that remained from the other assays was not sufficient to evaluate its acetylcholinesterase inhibition activity.

1,8-Cineole and limonene are the most important components of *E. globulus* oil. 1,8-Cineole has been reported as presenting a relative good AChE inhibition activity [38,39], therefore the relative good activity of *E. globulus* oil may be attributed to 1,8-cineole. However the IC_50_ value reported by those authors (0.06 mg/mL) for this component was lower; that is, the activity found was better than that found in the present work (0.1082 mg/mL). Limonene, the most important component found in the *E. globulus* oil, had a poorer activity (0.5863 mg/mL) than that of 1,8-cineole, but apparently not affecting the activity of the whole oil, since the IC_50_ values found for the oil (0.1298 mg/mL) and 1,8-cineole (0.1082 mg/mL) were not significantly different. *C. limon* oil which was practically entirely constituted by limonene had a lower capacity for inhibiting AChE (0.8499 mg/mL) than *E. globulus* oil and limonene. This component was even reported as being inactive by some authors [6].

The lowest anticholinesterase activity was found for *F. vulgare* oil (1.1877 mg/mL), mainly constituted by *trans*-anethole, which among the standards used, had also the lowest activity (IC_50_ = 1.3244 mg/mL). The IC_50_ value reported by some authors [40] for *trans*-anethole was somehow different to that, because they found for this phenylpropanoid a value 10 times lower (0.135 mg/mL). The same authors found that limonene also possessed acetylcholinesterase inhibition activity, nevertheless inferior to that of *trans*-anethole, that is, the opposite to what was found by us.

δ-3-Carene presented the best activity (IC_50_ = 0.0358 mg/mL). Such relative good activity was already reported elsewhere [41]. The authors found that this bicyclic monoterpene hydrocarbon was a strong inhibitor of AChE activity, reporting also the importance of the position of the double bond on the activity. Despite the presence of this component in a relative high percentage in *C. sempervirens* oil, it was not the best essential oil as inhibitor of AChE activity (Table 2).

Limonene, other major component of the oil with significant higher IC_50_, may have been responsible for the lowest activity of *C. sempervirens* oil. α-Pinene was assayed, but a turbidity present in the reaction mixture did not allow evaluation of its AChE inhibition activity. Nevertheless, this component, a bicyclic monoterpene has been also reported as possessing strong AChE inhibition activity [41]. Our results may therefore suggest an antagonistic effect among the components of the oil of *C. sempervirens*. On the other hand, the strongest activities of δ-3-carene and α-pinene may partly explain the best activity of that oil at higher concentrations than *E. globulus* oil, although this presented the lowest IC_50_ value.

*T. vulgaris* oil also presented capacity for inhibiting AChE activity (Figure 4A and Table 2). This may be attributed to its major components thymol and carvacrol. These two phenol monoterpenes were already reported as possessing such a property [42], being carvacrol more effective than thymol, as seen in the present work. The authors stress the importance of the position of the hydroxyl group in the molecular structure of those isomers on the AChE inhibitory activity. As for α-pinene, the IC_50_ value of *p*-cymene was not evaluated due to the turbidity presented by the reaction mixture.

In a recent publication [7] in which the essential oils of some Portuguese *Thymus* species were evaluated as potential AChE inhibitors showed that all carvacrol, borneol or 1,8-cineole-rich oils presented activity. Borneol used as standard in our work also showed a relative good activity (Figure 4A and Table 2).

## 3. Experimental

### 3.1. Samples

The essential oils were provided by Zaraphyt (Rabat, Morocco). According to the enterprise, the essential oils were obtained from the aerial parts of the plants, by steam distillation, except that of *C. limon* whose oil was obtained from the peel by expression.

### 3.2. Standards and Reagents

Thymol and 1,8-cineole were purchased from Montplet & Esteban SA (Barcelona, Spain). Linalool was purchased from Riedel-de-Haën Laboratory Chemicals (Seelze, Germany). α-Pinene was purchased from Merck-Schuchardt (Hohenbrunn, Germany). *p*-Cymene was purchased from VWR International (Oeiras, Portugal). 6-Hydroxy-2,5,7,8-tetramethylchroman-2-carboxylic acid (Trolox^®^), *trans*-anethole, borneol and linalyl acetate were purchased from Fluka Chemicals (Buchs, Switzerland). Limonene, acetylthiocholine iodide (ATCI), AChE (type VI-S) from the electric gel *Electrophorus electricus*, 5,5´-dithiobis(2-nitrobenzoic acid) (DTNB) were purchased from Sigma-Aldrich Chemie, (Steinheim, Germany).

### 3.3. Chemical Analysis of the Essential Oils

#### 3.3.1. Chemical Analysis of the Essential Oils by Gas Chromatography (GC)

Gas chromatographic analyses were performed using a Autosystem XL (Perkin Elmer, Shelton, CT, USA) gas chromatograph equipped with two flame ionization detectors (FIDs), a data-handling system and a vapourizing injector port, into which two columns of different polarities were installed: A DB-1 fused-silica column (30 m × 0.25 mm i.d., film thickness 0.25 μm; J&W Scientific, Rancho Cordova, CA, USA) and a DB-17HT fused-silica column (30 m × 0.25 mm i.d., film thickness 0.15 μm; J&W Scientific). Oven temperature was programmed from 45 °C to 175 °C at 3 °C/min, then at 15 °C/min to 300 °C, then held isothermal for 10 min; injector and detector temperatures were held at 280 °C and 300 °C, respectively; carrier gas, hydrogen, adjusted to a linear velocity of 30 cm/s. The samples were injected using the split sampling technique, ratio 1:50.

#### 3.3.2. Gas Chromatography-Mass Spectrometry (GC-MS)

The GC-MS unit consisted of a Autosystem XL (Perkin-Elmer) gas chromatograph, equipped with a DB-1 fused-silica column (30 m × 0.25 mm i.d., film thickness 0.25 μm; J&W Scientific) and interfaced with a Turbomass mass spectrometer (software v. 4.1, Perkin-Elmer). Injector and oven temperatures were as above; transfer line temperature, 280 °C; ion trap temperature, 220 °C; carrier gas, helium, adjusted to a linear velocity of 30 cm/s; split ratio, 1:40; ionization energy, 70 eV; ionization current, 60 μA; scan range, 40–300 u; scan time, 1 s. The identity of the components was assigned by comparison of their retention indices, relative to C_9_–C_21_* n*-alkane indices and GC-MS spectra from a home-made library, constructed based on analyses of reference oils, laboratory-synthesized components and commercially available standards. Only the main components whose concentrations were greater than 10% were considered in the present work. Standards of almost of these components were used for the evaluation of antioxidant activity and acetylcholinesterase inhibition.

### 3.4. Antioxidant Activities

#### 3.4.1. Thiobarbituric Acid Reactive Species (TBARS)

The ability of the oils and standards to inhibit malondialdehyde formation, and therefore lipid peroxidation, was determined by using a modified thiobarbituric acid reactive species (TBARS) assay. Egg yolk homogenates were used as a lipid-rich medium obtained as described elsewhere [25]. Briefly, 10% (w/v) homogenate (0.5 mL) and sample (0.1 mL) containing essential oil or standards, soluble in methanol, were added to a test tube and made up to 1 mL with distilled water. Then, 20% acetic acid (1.5 mL, pH 3.5) and 0.8% (w/v) thiobarbituric acid (TBA, 1.5 mL) in 1.1% (w/v) sodium dodecyl sulphate (SDS) were added. The resulting mixture was vortexed and heated at 95 °C for 60 min. After cooling, at room temperature, 1-butanol (5 mL) was added to each tube; the contents of the tubes were stirred and centrifuged at 3,000 rpm for 10 min. The absorbance of the organic upper layer was measured at 532 nm using a Shimadzu 160-UV spectrophotometer (Kyoto, Japan). All of the values were based on the percentage antioxidant index (AI%), whereby the control was completely peroxidized and each oil demonstrated a degree of change; the percentage inhibition was calculated using the formula (1 − *T*/*C*) × 100, where *C *is the absorbance value of the fully oxidized control and *T *is the absorbance of the test sample. The antioxidant capacity was determined from three replicates. The percentage antioxidant index was plotted against the concentrations of samples or standards and IC_50_ values were determined (concentration of essential oil or standard to prevent 50% of lipid oxidation).

#### 3.4.2. DPPH Free Radical-Scavenging Activity

Fifty microlitres of various concentrations (0.07–21 mg/mL) of samples or standards were added to 60 mM methanolic solution of DPPH (2 mL). Absorbance measurements were read at 517 nm, after 60 min of incubation time at room temperature (A_1_). Absorption of a blank sample containing the same amount of methanol and DPPH solution acted as the negative control (A_0_). The percentage inhibition *[(A_0_ − A_1_/A_0_) × 100]* was plotted against sample or standard content and IC_50_ was determined (concentration of essential oil or standard able to scavenger 50% of DPPH free radical).

#### 3.4.3. Reductive Potential

Each sample or standard was mixed with phosphate buffer (2.5 mL, 0.2 M, pH 6.6) and potassium ferricyanide [K_3_Fe(CN)_6_] (2.5 mL, 1%). The mixture was incubated at 50 °C for 20 min. A portion (2.5 mL) of trichloroacetic acid (10%) was added to the mixture, which was then centrifuged for 10 min at 3,000 rpm. The upper layer of solution (2.5 mL) was mixed with distilled water (2.5 mL) and FeCl_3_ (0.5 mL, 0.1%), and the absorbance was measured at 700 nm in a Shimadzu 160-UV spectrophotometer (Kyoto, Japan).

### 3.5. Acetylcholinesterase (AChE) Inhibitory Activity

AChE inhibitory activity was measured by using an assay described by Ellman *et al.* [43] along with the modifications described by Hammond and Forster [44]. Briefly, the oil sample (25 µL) was dissolved in ethanol along with the buffer (50 µL) and 0.22 U/mL AChE (25 µL), and the mixture was incubated at 37 °C for 15 min. The time at which the first enzyme addition was performed was considered as time zero. In this case, because the further analyses were performed using an end-point reading, the kinetics of reactions were not considered to be important. After the 15 min incubation, 3 mM DTNB (125 µL) and 15 mM ATCI (25 µL) were added, and the final mixture was incubated at room temperature for 30 min. The absorbance of the mixture was measured at 412 nm by using a microplate reader (Synergy H4 Hybrid Multi-Mode Microplate Reader, BioTek, Winooski, VT, USA). A control mixture was prepared by using 25 µL of ethanol instead of the oil sample, with all other procedures similar to those used in the case of the sample mixture. The percentage inhibition of enzyme activity was calculated by comparison with the negative control: *% = [(A_0_ − A_1_)/A_0_] × 100* where A_0_ was the absorbance of the blank sample and A_1_ was the absorbance of the sample. Tests were carried out in triplicate. Sample concentration providing 50% inhibition (IC_50_) was obtained plotting the inhibition percentage against essential oil or standard concentrations.

### 3.6. Statistical Analysis

Statistical comparisons were made with one-way ANOVA followed by Tukey multiple comparison test. The level of significance was set at *P < 0.05*. Statistical calculation was performed using PASW Statistics, Version 18 (2009).

## 4. Conclusions

The essential oil of *T. vulgaris* presented good antioxidant activity as measured by the TBARS, DPPH and reductive potential methods. Thymol and carvacrol may be the components responsible for such activity. Nevertheless, the reductive potential of some standards, namely of that of δ-3-carene, was also remarkable, although unexpected. This may be due to the autoxidation of this compound under the experimental conditions used. That compound was the most effective as an AChE inhibitor. Nevertheless the most effective oil, that is, the one with the lowest IC_50_ value, was that of *E. globulus* in which 1,8-cineole and limonene predominated. In spite of this low IC_50_, the oils of *C. sempervirens* and *T. vulgaris* at higher concentrations were better inhibitors than *E. globulus*.
